# First-aid training for primary Healthcare providers on a remote Island: a mixed-methods study

**DOI:** 10.1186/s12909-024-05768-6

**Published:** 2024-07-23

**Authors:** Ninh Do Thi, Giang Hoang Thi, Yoonjung Lee, Khue Pham Minh, Hai Nguyen Thanh, Jwa-Seop Shin, Tuyen Luong Xuan

**Affiliations:** 1 College of Nursing, Ewha Womans University, Seoul, Korea; 2 Hai Phong University of Medicine and Pharmacy, 72 Nguyen Binh Khiem, Hai Phong, Vietnam; 3 Department of Medical Education, Seoul National University College of Medicine, Seoul, Korea; 4 Vietnam National Institute of Maritime Medicine, Hai Phong, Vietnam

**Keywords:** Mixed-methods, First aid, Continuous training, Primary healthcare provider, Vietnam

## Abstract

**Background:**

Ensuring ongoing first-aid training for primary healthcare providers (PHPs) is one of the critical strategies for providing quality health services and contributing to achieving universal health coverage. However, PHPs have received insufficient attention in terms of training and capacity building, especially in the remote areas of low-to-middle-income countries. This study evaluated the effectiveness of a first-aid training program for PHPs on a Vietnamese island and explored their perspectives and experiences regarding first-aid implementation.

**Methods:**

A mixed-methods study was conducted among 39 PHPs working in community healthcare centers. The quantitative method utilized a quasi-experimental design to evaluate participants’ first-aid knowledge at three time points: pre-training, immediately post-training, and three months post-training. Sixteen of the PHPs participated in subsequent semi-structured focus group interviews using the qualitative method. Quantitative data were analyzed using repeated measures analysis of variance (ANOVA), while qualitative data were subjected to thematic analysis.

**Results:**

The quantitative results showed a significant improvement in both the overall mean first-aid knowledge scores and the subdimensions of the first-aid knowledge scores among healthcare providers post-training. There was a statistically significant difference between the baseline and immediate posttest and follow-up knowledge scores (*p* < 0.001). However, the difference in knowledge scores between the immediate posttest and three-month follow-up was not significant (*p* > 0.05). Three main themes emerged from the focus group discussions: perception of first-aid in remote areas, facilitators and barriers. Participants identified barriers, including infrastructure limitations, shortage of the primary healthcare workforce, inadequate competencies, and insufficient resources. Conversely, receiving considerable support from colleagues and the benefits of communication technologies in implementing first aid were mentioned as facilitators. The training bolstered the participants’ confidence in their first-aid responses, and there was a desire for continued education.

**Conclusions:**

Implementing periodic first-aid refresher training for PHPs in a nationwide resource-limited setting can contribute significantly to achieving universal health coverage goals. This approach potentially enhances the preparedness of healthcare providers in these areas to deliver timely and effective first aid during emergencies, which may lead to more consistent primary healthcare services despite various challenges.

**Supplementary Information:**

The online version contains supplementary material available at 10.1186/s12909-024-05768-6.

## Introduction

The World Health Organization (WHO) has determined that Universal Health Coverage is a fundamental goal for strengthening healthcare systems and achieving better health outcomes worldwide [1]. Universal Health Coverage ensures that all people have access to quality health services without the financial hardships associated with paying for care [2]. Primary healthcare (PHC) plays a crucial role in attaining universal health coverage because the majority of healthcare needs can be met through PHC interventions [3]. Therefore, strengthening the quality of PHC at the grassroots level is essential for all countries. Additionally, the WHO emphasizes the central role of the primary health workforce in emergency situations, life-threatening responses, and injury management, along with highlighting the importance of training these frontline staff [4]. However, PHPs have received insufficient attention in terms of training and capacity building, particularly in the remote areas of low- and middle-income countries, including in Vietnam [5].

Vietnam’s healthcare system consists of public and private healthcare sectors, with the public system predominantly providing preventive and curative healthcare services to the population [6]. The public healthcare system is classified into four hierarchical levels with different roles and functions [6]. The two higher levels, central and provincial, deliver tertiary and secondary healthcare services through specialized hospitals and professionals [7]. The two lower levels, district and commune, provide PHC services via healthcare centers that function as the foundation of the national health system and contribute significantly to implementing the nation’s health program [7, 8]. Although PHC services at health centers are free or low-priced for the nationwide population with public health insurance, many people skip these facilities and reach provincial or central hospitals expecting higher-quality healthcare services [9]. Providing health services through less-qualified health staff has accounted for these issues at health centers for decades, primarily because of inadequate professional knowledge and clinical practice skills [9, 10]. These challenges are particularly evident in the rural and remote communities [11]. Literature indicates that contextual factors, such as geographic conditions and limited resources, contribute to these issues, creating substantial barriers to accessing healthcare, supporting a skilled workforce, and implementing training programs at the grassroots level in Vietnam [5].

Providing first-aid training to healthcare providers at community health centers is one of the initial steps to compensate for these deficiencies in primary healthcare workforce development [12]. First-aid training equips PHPs with the necessary knowledge and skills to respond immediately and effectively to sudden, life-threatening, or common emergencies, thereby increasing the likelihood of successful treatment and safe living for residents [13]. Although previous studies have focused on first-aid training, most concentrated on the effectiveness of cardiopulmonary resuscitation in various subjects without focusing on healthcare providers [14–17]. Moreover, other critical first-aid skills, such as choking, burning, stroke, trauma, and hemorrhage, have rarely been studied [13, 18]. Furthermore, healthcare providers repeatedly encounter challenges associated with inadequate basic lifesaving skills, insufficient training, limited opportunities for practice and skill development, and inefficient and short retention of skills [19–22] that may reduce healthcare quality. Importantly, first-aid training programs specifically designed for PHPs in remote areas, particularly islands, are lacking. Therefore, developing a tailored first-aid training program for PHPs in resource-limited settings is necessary to enhance their knowledge and skills and ultimately improve the quality of primary care.

**Fig. 1:**
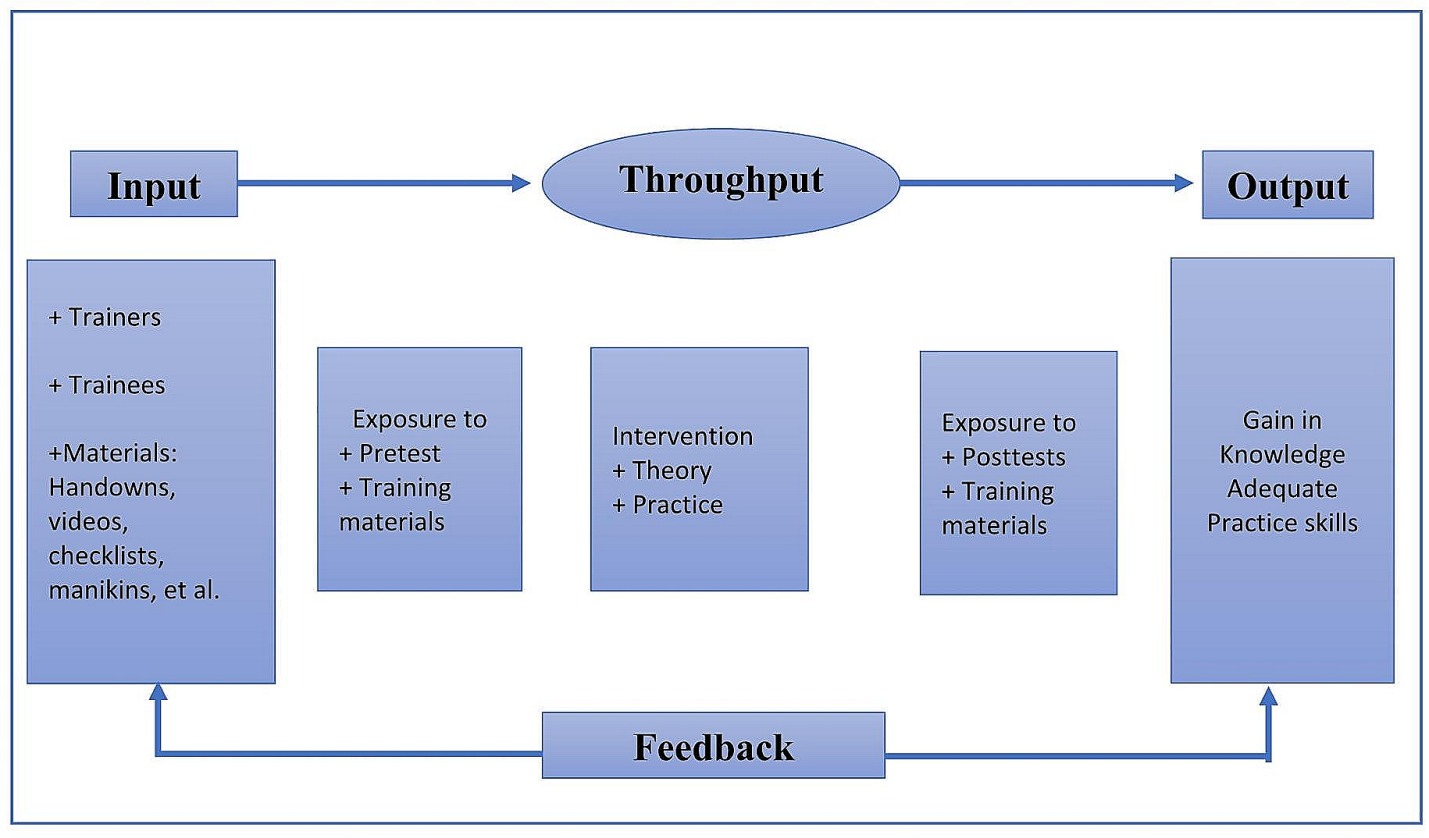
Conceptual framework based on General Sytem Theory of Ludwig Von Bertalanffy (1986)

This study aimed to evaluate the effectiveness of first-aid training programs for PHPs on a remote island in Vietnam. Additionally, we sought insights into the experiences and perceptions of island PHPs regarding the implementation of first aid. These findings provide evidence supporting the expansion of comprehensive first-aid training programs for PHPs in remote areas, both nationally and in low-to-middle-income countries. The conceptual framework for this study was developed based on the General Systems Theory [23, 24], which comprises three elements: input, throughput, and output (Fig. 1).

## Methods

### Study design

This study used a mixed-methods approach. Quantitative data were collected using a quasi-experimental design to investigate the effectiveness of the first-aid training program for PHPs. Qualitative data were obtained through group interviews to gain insights into the perceptions and experiences of PHPs regarding the implementation of first aid.

### The quantitative study

#### Setting and participants

This study was conducted in an island district in northern Vietnam. The district has an area of 325,6 sq. km, 12 communities, and approximately 32,090 residents. The healthcare system in the island district is similar to that in other provinces throughout Vietnam; however, differences in geographical and settlement conditions have resulted in remarkable disparities in health needs between the island and mainland populations and limited access to diverse health services for the island population. Healthcare providers encounter various challenges in delivering healthcare services to island residents.

The participants were PHPs (nurses, midwives, and physicians) in community health centers recruited using convenience sampling. PHPs were eligible to participate if they directly provided healthcare services in a healthcare setting, completed the theoretical and practical training program, and participated in the data collection before and after the program. A recruitment letter was sent to all thirteen community health centers on the island to recruit participants, and the sample size was reached according to the order of registration. The sample size was calculated using the G* Power 3.1.9.7. program with an effect size of 0.25, alpha level of 0.05, correlation among repeated measures of 0.5, and power of 0.90. Accordingly, the required sample size for the repeated-measures analysis of variance (ANOVA) was 36. To account for potential dropouts during the follow-up period, 39 PHPs with at least one year of experience voluntarily participated in the training.

#### The intervention

The first-aid training program was conducted face-to face over two days. The educational content comprised theoretical and practical training covering five dimensions: basic life support, drowning, bleeding, fractures, and burns. The theoretical components were delivered through didactic lectures, videos, and case studies. For practical training, 39 participants were divided into seven groups, each consisting of five people and one group comprising four people. Each group engaged in hands-on practice of first-aid procedures using manikins, learning devices, and equipment. Lecturers with experience teaching first aid at hospitals and medical universities guided and supported the participants throughout the training sessions. The participants were provided books, videos, audio materials, and slides two weeks in advance to enhance their teaching and learning outcomes. They were expected to review and study these resources before the training began. After training, first-aid skills were immediately evaluated using scenario-based assessments and checklists. This ensured that all participants were adept at and ready to apply first-aid techniques in real-life emergencies.

#### Instruments

Based on a needs assessment of first-aid training among island Primary Health Practitioners (PHPs) and a synthesized review of first-aid literature, a questionnaire was developed. This tool is organized into two parts to collect participants’ demographic information and assess their knowledge across five dimensions covered during the training. The first part included demographic information, such as age, gender, job titles, participation in a previous first-aid program, and working experience. The second part consisted of 30 multiple choice questions on knowledge of first aid, including nine items on fractures, seven on basic life support, seven on bleeding, four on drowning, and three on burns, with a higher score indicating a better level of first aid knowledge (Supplementary file 1). The questions specifically focused on common emergencies encountered by PHPs in communities and assessed the participants’ understanding of appropriate responses to these emergencies. The questionnaire content validity includes the item content validity index (I-CVI), and the overall scale content validity index (S-CVI), which was analyzed based on the proportion of ratings from a expert panel. The I-CVI value of each item ranged from 0.67 to 1.00, and the S-CVI was 0.97, indicating that the scale had good content validity. The reliability of the first aid scale was assessed using KR20, was 0.75. Therefore, the instrument demonstrated sufficient reliability and validity in this study.

#### Data collection and analysis

Participants’ knowledge of first aid was evaluated at three different time points: before training (pretest), immediately after training (posttest), and three months later (follow-up). Data were collected between September 2022 and May 2023. The collected data were analyzed using SPSS version 26.0. Descriptive statistics were used to calculate the mean (SD) for continuous variables and frequencies for categorical variables. Friedman’s ANOVA test was employed to assess within-subject differences in participants’ knowledge between the pretest, posttest, and follow-up. Statistical significance was defined as a p-value less than 0.05.

### The qualitative study

The qualitative study employed group interviews led by researchers experienced in qualitative research methods. Sixteen PHPs from the quantitative sample willing to participate in the interview were organized into three focus groups. The first two groups contained five participants, whereas the third comprised six. Each interview was conducted immediately after the training session, ranging from 45 to 60 minutes. With the consent of the participants, the interviews were audio-recorded and transcribed verbatim to ensure the accuracy of the analysis. The interview questions included “ How do you perceive the implementation of first aid in your area?“, “How did you handle first-aid situations in your center?” and “What factors influence your first-aid implementation?“. The data were analyzed using the content analysis approach described by Elo and Kyngas [25]. After each session, the interview contents were transcribed word-for-word to retain accuracy and prevent distortions or misinterpretations. The researcher repeatedly read the interview transcripts to become familiar with the context. A meticulous, line-by-line examination pinpointed meaningful units, ranging from single words to whole sentences, that convey distinct insights from the interview data. After identifying these units, subsequent discussions among the authors categorized them into larger units. Finally, overarching themes were derived and presented as barriers and facilitators of implementing first-aid on the island.

### Ethics

This study was approved by the Research Ethics Committee of the Haiphong University of Medicine and Pharmacy (Approval No. 01/HDDD). Written consent for the participation and recording of interviews was obtained from the trainees.

## Results

### Participant characteristics

Thirty-nine PHPs participated in training and completed the pretest, posttest, and follow-up test. The results revealed that the participants had a mean age of 38.77 years (SD = 7.93), and 61.5% were female. Additionally, 61.5% of the participants had more than nine years of experience, while 76.9% had previously attended courses related to first aid. Additionally, from the initial pool of 39 participants, 16 trainees voluntarily participated in the group interviews. Details of the participants are presented in Table 1.

**Table 1:** Participant characteristics (*N* = 39)

Characteristic	*N*	%	Mean	SD	Range
Age, years			38.77	7.93	29–59
Gender					
Male	15	38.5			
Female	24	61.5			
Experience					
< 5 years	3	7.7			
5–9 years	12	30.8			
> 9 years	24	61.5			
Job titles					
Nurses	14	36			
Midwives	9	23			
Physicians	16	41			
Previous first-aid training					
Yes	30	76.9			
No	9	23.1			

### Results of the quantitative components

**Fig. 2:**
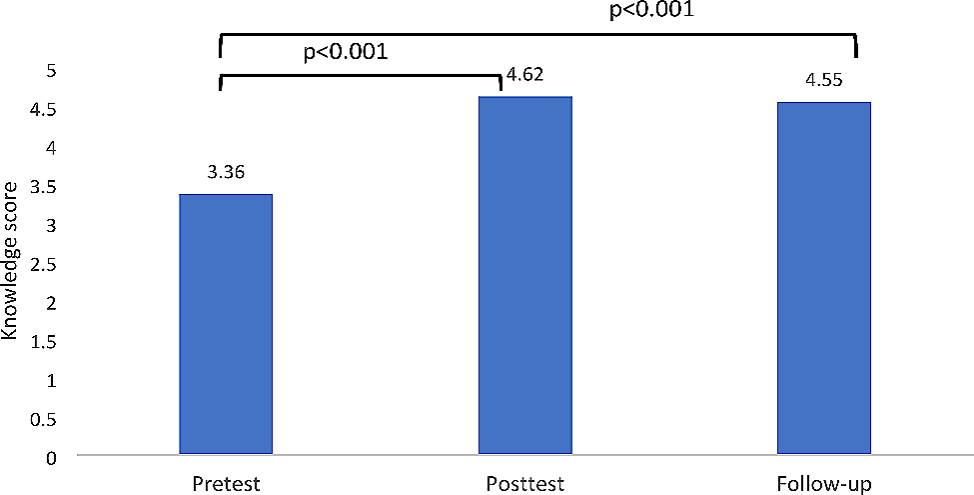
Comparing knowledge scores pretest/posttest, pretest/follow-up, and posttest /follow-up (*N* = 39)

The results indicated a significant improvement in the overall mean knowledge scores after the training (*p* < 0.001). Specifically, there was a statistically significant difference between the baseline knowledge score and both the immediate posttest and follow-up knowledge scores, with *p* < 0.001. However, no significant difference was observed in knowledge scores between the immediate posttest and follow-up (*p* > 0.05) (Fig. 2).

This study evaluated the knowledge scores across five first-aid dimensions: basic life support, bleeding, fractures, drowning, and burns. Friedman’s ANOVA demonstrated a significant increase in knowledge scores across all dimensions after the intervention (*p* < 0.001). Specifically, the immediate posttest scores for basic life support and burns surged significantly from baseline, although they were marginally lower than the scores at the three-month follow-up (*X*^*2*^ = 31.70, *p* < 0.001; *X*^*2*^ = 37.48, *p* < 0.001, respectively). Similarly, the immediate posttest scores for bleeding, fracture, and drowning saw a significant rise compared to pretest scores, but they edged out the scores at the follow-up (*X*^*2*^ = 41.90, *p* < 0.001; *X*^*2*^ = 42.24, *p* < 0.001; *X*^*2*^ = 25.29, *p* < 0.05, respectively) (Table 2).

**Table 2:** Differences in knowledge scores at pretest, posttest, and three-month follow-up across five dimensions (*N* = 39)

**Knowledge**	**Pretest**		Posttest		Follow-up		X^2^	Gain Pre-Post (%)	Gain Pre-Follow (%)	*p*
	Mean	SD	Mean	SD	Mean	SD				
BLS	3.44	1.39	4.72	.50	5.00	1.32	31.70	37.20	45.34	< .001
Bleeding	5.03	1.35	6.64	.47	6.33	.77	41.90	32.00	25.84	< .001
Fracture	5.08	1.45	7.46	1.21	6.79	1.26	42.24	46.85	33.66	< .001
Downing	3.03	.74	3.41	.71	3.21	.69	25.29	12.54	6.00	< .005
Burn	.28	.46	.49	.55	1.23	.63	37.48	75.00	339.28	< .001
Total	3.36	.70	4.62	.36	4.55	.64	52.00	37.50	35.41	< .001

BLS: basic life support

### Results of the qualitative components

Three main themes emerged during the data analysis, including perception of first-aid in remote areas, barriers and facilitators, which represented the actual issues in delivering first aid on the island by PHPs.

### The perception of first-aid in remote areas

Primary healthcare providers recognize the critical role of first aid as a first line of response in medical emergencies. Due to the potential delays in accessing advanced medical care in remote areas, first aid not only becomes a crucial skill set but also a vital component of community health strategy. They commonly express a strong belief in the need for comprehensive first aid training that is regularly updated and tailored to the specific challenges and risks of their community. They highlight the relevance of first aid in their daily practice, noting its impact on patient outcomes and community confidence in the healthcare system. There is a prevalent attitude that effective first aid training enhances their capability to manage emergencies more efficiently and provides a buffer time that can be critical for patient survival before professional medical help arrives.

*In remote healthcare*, *first aid isn’t just an added skill—it’s often the difference between life and death. We are the first and sometimes the only line of defence when an emergency strikes. Regular*, *hands-on training in first aid is essential for us to keep up with the best ways to respond…*.

…Everyone in healthcare here knows that these skills are absolutely crucial. We need training that considers the specifics of our settings—like dealing with sea injuries, *downing or snake bites*, *which are common in our area*.

*…The training we receive has to mirror the realities we face—long distances*, *limited resources*, *and high reliance on each other’s skills. Enhanced first aid training that’s recurrent and comprehensive is not negotiable; it’s vital*.

### Barriers

Participants identified geographic location and transportation infrastructure as substantial barriers to accessing emergencies and transferring patients to advanced medical facilities. As there is no public transportation on the island, calling an ambulance increases the waiting time for patients to receive healthcare delivery from a higher level of care. Therefore, PHPs utilize the available vehicles to manage patient transfers and minimize delays in reaching advanced care facilities.

*…moving to the mainland is difficult. For example*, *many days there are storms and winds*, *and transporting patients across the ferry and over the ship is terrifying. The boats are tiny*, *but we still had to transfer the patient.*

*It takes time to wait for an ambulance*, *so we often transfer directly to the district center…*.

In addition to transportation, deficiencies and maldistribution of resources, such as financial resources, necessary equipment, and medications, contribute to ineffective first aid. Many healthcare centers have insufficient medical devices and essential medicines listed in the insurance drug formulary.

*The government also provides equipment*, *but it is still very poor and small number*, *not able to provide many things. Also sometimes*, *we apply to get equipment from the agency*, *but the agencys’ funding is still limited.*

*Most patients who received first aid or emergency*, *such as trauma or fracture*, *did not return after being transferred; the medical equipment used during the initial treatment is lost*, *and we have to purchase it again using our own money.*

According to most participants, severe shortages of human resources and inadequate skills and knowledge were identified as significant barriers to healthcare and the implementation of first-aid. The lack of practical training programs and impractical working conditions due to the limited number of patients were cited as reasons for the staff’s insufficient competencies.

*Due to low wages and isolated locations*, *fewer people want to work here*, *leading to a lack of staff. Many night shifts have only one staff member*, *so when there is an emergency*, *the staff cannot handle it…. I was only trained when studying at medical school and have not received any further training since. Some short courses were not related to first aid….*

*In a case of fall*, *the staff only injected the patient and sent him home. However*, *the patient’s family took him to a higher level because of concerns about his condition. He died before reaching the hospital because of internal injuries sustained during the fall…*.

*Here*, *just few cases a month*, *they bypass the community to go directly to the city…*.

### Facilitators

The participants highlighted the most notable advantage of companionship from colleagues and upper-level staff who readily responded and provided support in times of necessity.

*It was 10 p.m.*, *and I was alone at the health station; the patient came because of a thigh fracture after an accident. I called my colleague to help. We gave the patient first aid and then moved him to the city hospital. My colleague stayed on duty.*

*Last time I had to call the director of the district healthcare center to ask for advice on curing a patient with a neck fracture*, *he provided his personal phone number as a hotline.*

Participants also emphasized the importance and convenience of communication technologies in connecting patients, PHC providers, and higher healthcare levels. Patients can contact community staff beforehand in cases of unexpected situations, and primary healthcare staff can quickly seek consultation from provincial or district doctors for treatment guidance through mobile phones.

*When we received a call from a patient or their relatives informing of an accident*, *we immediately went to the scene or prepared to pick the patient up at the health center*.

Crucially, all participants expressed increased confidence in handling emergencies after the training. Additionally, they expressed a wish to undergo regular first-aid training every three to six months to update their knowledge. They showed interest in various types of first-aid training, with an emphasis on procedures specific to coastal regions, such as poisoning, snakebites, and patient transport via boats and ships. Furthermore, they expected all PHPs at island health centers to receive regular training.

*After training*, *I am more confident and can teach other staff in my centers…*.

*To be honest*, *we would like to study many things because of the lack of opportunities to update knowledge; if possible every three to six months*, *every staff receives training is good…*.

*We want to learn more about typical first aid here*, *for example*, *poisoning*, *snakebite*, *patient transportation by boats*, *ships…*.

## Discussion

This mixed-methods study investigated the effectiveness of a first-aid training program that aimed to improve PHPs’ knowledge and explore their perceptions and experiences regarding implementing first-aid on a remote Vietnamese island. The quantitative results showed a significant increase in the total and sub-dimensional mean knowledge scores of PHPs after training. The qualitative data revealed threemain themes: perception of firs-aid in remote area, barriers and facilitators.

In this study, the mean knowledge scores among healthcare providers significantly increased after training, which is consistent with findings from previous studies conducted on different subjects [19, 20, 22, 26]. Despite the differences in research subjects across these studies, the results are comparable as they all underwent a first-aid learning experience [27].

The study identified a significant difference between the baseline and knowledge scores obtained in the posttest and after the three-month follow-up. However, no statistically significant difference in knowledge scores was observed between the post-test and the three-month follow-up, although the mean knowledge score slightly decreased after three months of training. This finding contrasts with an intervention conducted on PHPs in the USA [28], and other previous studies [29, 30] on different research subjects, which showed substantial deterioration of knowledge after initial training. This disparity could be explained by the extended evaluation period, which is longer than the duration of our study. This result emphasizes the need for repeated interventions or follow-ups to help maintain the knowledge gained during training sessions.

The qualitative analysis revealed that primary healthcare providers in remote areas perceive first aid as a critical component of their response to medical emergencies, given the frequent delays in accessing advanced care. They emphasize the necessity of comprehensive, regularly updated first aid training tailored to their specific community challenges, such as sea injuries, drownings, and snake bites. This training significantly impacts patient outcomes and enhances community confidence in the healthcare system. The insights suggest that any intervention aimed at improving primary healthcare in remote areas should prioritize context-specific first aid training to ensure healthcare providers are well-prepared for the unique emergencies they face, ultimately improving patient outcomes [12].

Like other low- and middle-income countries, the Vietnamese primary healthcare system has been facing numerous challenges that impede the quality of healthcare services, including a lack of workforce and competencies, and limitations in infrastructure and resources [9, 31]. This situation becomes even more complex in remote and island areas. The Ministry of Health’s Joint Annual Health Review [32] has highlighted the unequal distribution of financial resources between higher- and primary-level care, with insufficient investments in PHC to meet the demands. Vietnam has recently been striving towards achieving UHC through national programs aimed at strengthening and improving the quality of primary care, with a specific focus on enhancing infrastructure and staff competence at the grassroots level [7, 8]. Although community health centers have a higher capacity to provide health services, UHC has not yet been fully realized [33].

A lack of human resources and competencies is related to geographic locations, low salaries, and inadequate training and promotion opportunities, which prevent young healthcare professionals from entering and engaging in the primary healthcare system. Issues related to recruitment and retention of the primary healthcare workforce have been documented in the literature in Vietnam and other countries worldwide [5, 9, 34, 35]. Moreover, PHC providers’ competency deficiency might be explained by the recent reduction in the number of patients examined at health centers, which has led to a lack of clinical practice experience. Patients bypass primary healthcare centers to reach secondary and tertiary hospitals, which are expected to provide higher-quality healthcare services. The Ministry of Health [32] revealed that 54–65% of patients seeking care at central hospitals have health conditions that can be diagnosed and treated at lower levels of care. Furthermore, our findings indicated that one-fourth of the participants had not been retrained in first aid since graduating from medical school despite serving in the healthcare sector for almost nine years of mean working experience. This suggests a potential requirement for periodic refresher training for PHPs.

Regardless of the barriers to first aid, PHPs received support from their colleagues and benefited from communication technologies. In emergencies, teamwork and interprofessional collaboration play crucial roles in ensuring effective PHC through interactive efforts, communication, respect, and support among team members [36–38]. Therefore, it is essential to continually emphasize collaboration and inter-professional training in first-aid programs for PHPs. Furthermore, the PHC staff confirmed the usefulness of communication technologies in carrying out first aid. This finding aligns with previous studies that have demonstrated the crucial role of technologies in healthcare, as they serve as the foundation for services aimed at preventing, diagnosing, and treating illness and diseases, thereby enhancing the quality and safety of healthcare delivery systems [39, 40]. However, our findings revealed that PHC staff primarily relied on smartphones for communication during emergencies, modern technologies, such as remote consultations, digital platforms for data sharing, digital non-invasive care, and interconnected medical decision support, are extensively and broadly used as tools to deliver a wide range of services to remote areas and PHC settings in many countries [41]. They also include continuous education, lifelong learning, and telehealth [39]. Therefore, periodic first-aid refresher training for PHC staff, using modern approaches that combine both online and offline methods, can offer diverse content while ensuring training quality and cost-effectiveness.

PHPs recognized the importance of periodic first-aid refresher training and were willing to pursue new training opportunities to improve their competencies. They also emphasized the significance of regular training for all island staff to ensure quality care for islanders. Previous studies have underscored the necessity of continuing professional development opportunities for healthcare workers to enhance the quality of the work environment [42]. Investment in knowledge and skill development has been linked to increased retention [43], higher job satisfaction [44], and improved self-confidence among staff nurses [45]. These positive outcomes can lead to reduced vacancy rates and increased retention [46], that is one of the key strategies for achieving UHC in remote areas.

## Conclusions

The findings of this study highlight the importance of periodic first-aid refresher training. In addition to common first-aid skills, it emphasizes specific first-aid procedures tailored to coastal areas and interdisciplinary collaborative training to enhance the effectiveness of emergency responses and ensure the quality and safety of primary healthcare services. The study also emphasizes the need to address the challenges related to recruiting and retaining the primary healthcare workforce to achieve universal health coverage. Overall, this study provides valuable insights for policymakers, healthcare providers, and educators to enhance the quality and accessibility of PHC services in Vietnam and other low- and middle-income countries.

### Limitations

This study has several limitations. First, while participants were confident and ready to apply first-aid techniques in real-life emergencies after immediate evaluation following the training, this study did not assess the first-aid skills at baseline. Further research should evaluate the participant’s skills and attitudes before and after the intervention. Additionally, utilizing an existing first-aid self-efficacy instrument would aid in predicting trainees’ behavior in certain situations. Second, the follow-up period was only three months, which may not be sufficient to assess the long-term impact of the first-aid training program. Third, this study was conducted in the specific context of an isolated island in Vietnam, which may limit the generalizability of the findings to other settings. Fourth, self-administered measures may introduce the potential for social desirability response bias. Finally, there may be potential for errors and misunderstandings resulting from the primary author’s cultural background and language limitations. However, this risk was minimized through an iterative research process and by collaborating with colleagues involved in various stages of the study, including developing study materials, collecting data, interpreting participants’ input, and co-authoring this paper.

### Electronic supplementary material

Below is the link to the electronic supplementary material.

Supplementary Material 1

## Data Availability

Data are available upon request from the author at the following email dtninh90@gmail.com.

## Abbreviations

PHC: Primary healthcare
PHPs: Primary healthcare providers
WHO: World Health Organization
USA: United States of America
UHC: Universal Health Coverage
