# Supplementary material for: First-aid training for primary Healthcare providers on a remote Island: a mixed-methods study
Source: BMC Med Educ. 2024 Jul 23;24:790. doi: 10.1186/s12909-024-05768-6 (PMC11267758; doi:10.1186/s12909-024-05768-6)
Supplement: Supplementary file 1 — Supplementary Material 1 [file 12909_2024_5768_MOESM1_ESM.docx]

**Supplementaty file 1: Questions for first-aid knowleage**

1. **How should ice be applied to a fracture site?**

A. Directly on the skin

B. Wrapped in a cloth

C. With a heating pad

D. Not at all

1. **Where should chest compressions be performed during CPR?**

A. Lower half of the sternum

B. Lower third of the sternum

C. Middle half of the sternum

D. Upper half of the sternum

1. **Which type of burn affects only the outer layer of skin?**

A. First-degree burn

B. Second-degree burn

C. Third-degree burn

D. Fourth-degree burn

1. **What is the correct sequence in the Basic Life Support (BLS) protocol?**

A. A(airway) – B(breathing) – C(circulation)

B. B(breathing) – A(airway) – C(circulation)

C. C(circulation) – A(airway) – B(breathing)

D. B(breathing) – C(circulation) – A(airway)

1. **What should you do before moving a person with a suspected fracture?**

A. Align the fracture

B. Apply ice directly

C. Immobilize the fracture

D. Elevate the limb

1. **How should a burn be covered after cooling?**

A. With a wet cloth

B. With butter or oil

C. With a clean, dry cloth or dressing

D. With plastic wrap

1. **Which sign indicates that a tourniquet is too tight?**

A. Continued bleeding

B. Loss of limb sensation

C. Slow, steady blood flow

D. Bruising around the wound

1. **What is the first step in controlling severe external bleeding?**

A. Apply a tourniquet

B. Apply direct pressure

C. Clean the wound

D. Elevate the limb

1. **If a drowning victim is not breathing, what should you do first?**

A. Start CPR with rescue breaths

B. Perform the Heimlich maneuver

C. Check for external injuries

D. Call for advanced medical help

1. **How deep should chest compressions be for an adult during CPR?**

A. At least 3 centimeters

B. At least 4 centimeters

C. At least 5 centimeters

D. At least 6 centimeters

1. **What is the primary concern when treating a closed fracture?**

A. Realigning the bone

B. Immobilizing the fracture

C. Applying heat

D. Ignoring it

1. **Which type of bleeding is characterized by bright red, spurting blood?**

A. Venous bleeding

B. Arterial bleeding

C. Capillary bleeding

D. Internal bleeding

1. **What is the recommended compression-to-ventilation ratio for one-rescuer CPR on an adult?**

A. 15 compressions to 1 breath

B. 30 compressions to 2 breaths

C. 35 compressions to 3 breaths

D. 40 compressions to 4 breaths

1. **After pulling a drowning victim out of the water, what should you do next?**

A. Check for breathing

B. Perform abdominal thrusts

C. Wrap the victim in a blanket

D. Move the victim to a warm area

1. **How should a tourniquet be applied to control severe bleeding?**

A. Directly on the wound

B. 5-7 centimeters above the wound

C. 12-15 centimeters above the wound

D. 2.5 centimeters below the wound

1. **When should an Automated External Defibrillator (AED) be used during a cardiac emergency?**

A. Immediately upon arrival

B. After 2 minutes of CPR

C. Only by a medical professional

D. Only if CPR is unsuccessful after 5 minutes

1. **What is the optimal rate of chest compressions during CPR?**

A. 80-100 compressions per minute

B. 90-110 compressions per minute

C. 100-120 compressions per minute

D. 120-130 compressions per minute

1. **What should be the first step in treating a burn?**

A. Apply oil to the burn

B. Run cool water over the burn

C. Cover the burn with a wet cloth

D. Pop any blisters that form

1. **What should be avoided when treating a fracture?**

A. Applying a splint

B. Moving the patient

C. Keeping the limb immobile

D. Seeking medical attention

1. **What is the primary purpose of rescue breaths for a drowning victim?**

A. To prevent hypothermia

B. To remove water from the lungs

C. To provide oxygen to the victim

D. To calm the victim

1. **What is a compound fracture?**

A. A fracture with no open wound

B. A fracture that breaks through the skin

C. A minor crack in the bone

D. A dislocated joint

1. **Which of the following is true about compound fractures?**

A. They are less dangerous than closed fractures

B. They involve an open wound and risk of infection

C. They do not need medical attention

D. They can be treated with home remedies

1. **How can you confirm a fracture?**

A. By feeling the bone

B. By visual inspection

C. By using an X-ray

D. By patient’s complaint only

1. **In case of suspected cardiac arrest, which pulse should be checked to confirm the condition?**

A. Radial artery

B. Carotid artery

C. Popliteal artery

D. Femoral artery

1. **Why should a fractured limb be elevated?**

A. To increase blood flow

B. To reduce swelling

C. To help in realignment

D. To cause more pain

1. **What is the maximum time a tourniquet should remain in place?**

A. 1 hour

B. 2 hours

C. 4 hours

D. 6 hours

1. **What should be done after applying a pressure bandage to a wound?**

A. Check the patient's pulse below the bandage

B. Elevate the limb

C. Apply ice to the wound

D. Remove the bandage to inspect the wound every 10 minutes

1. **How should a spinal fracture be handled?**

A. By moving the patient carefully

B. By immobilizing and avoiding movement

C. By aligning the spine

D. By applying heat

1. **What is the first step when rescuing a drowning victim?**

A. Start chest compressions

B. Ensure the scene is safe

C. Give rescue breaths

D. Check for a pulse

1. **What is the correct procedure for cleaning a wound before dressing it?**

A. Use alcohol

B. Use hot water

C. Use clean water and soap

D. Use hydrogen peroxide
